# Lessons from cholera & *Vibrio cholerae*

**Published:** 2011-02

**Authors:** Asoke C. Ghose

**Affiliations:** Department of Microbiology, Bose Institute, Kolkata, India

**Keywords:** Cholera, cholera toxin, ORS, vaccine, VBNC, *Vibrio cholerae*

## Abstract

Cholera is an acute form of diarrhoeal disease that plagued human civilization over the centuries. The sudden and explosive onset of the disease in the form of an outbreak or epidemic, coupled with high mortality and morbidity rates, had a tragic impact on the personal as well as social life of people living in the affected areas. The enormity of human sufferings led clinicians and scientists to carry out extensive research on cholera and *Vibrio cholerae* (the causative bacterium of the disease) leading to major discoveries that opened up novel areas of research or new disciplines in biomedical sciences. An attempt is made here to summarize some of these breakthroughs and outline their significance in broader perspectives. Finally, the possible impact of the global socio-political scenario on the spread of cholera epidemics (pandemicity of cholera) is briefly discussed.

## Introduction

Cholera is an acute type of diarrheal illness that affected millions of people around the world over the centuries. Historically speaking, there are very few diseases that can match cholera in terms of its severity and explosive onset in the form of an outbreak or epidemic. Further, high mortality and morbidity rates associated with classical cholera had a tremendous tragic impact on the personal as well as social life of people living in the affected areas. As a consequence, the disease had found its place in the contemporary literary works in a number of instances where subtle intricacies of human relationship were craftily dealt with in the backdrop of an ongoing epidemic^1^–^3^.

The enormity of human sufferings also led clinicians and scientists to carry out extensive research to formulate effective treatment of cholera patients as well as to develop strategies for the prevention of the outbreak of the disease. As a result, considerable progress was made toward the goal through generation of novel information on the physiology, genomics and evolution of *Vibrio cholerae* (the causative bacterium of the disease), the mechanism of its survival within the host as well as in the environment, the transmission cycle of cholera, the pathophysiological and immunological aspects of host –bacterium interaction, *etc*. Some of the discoveries were of such profound importance that these opened up novel areas of research or did herald new disciplines in biomedical science, particularly those related to infectious diseases. An attempt is made here to summarize some of these breakthroughs that were achieved through studies on cholera or *V. cholerae* and outline their significance in broader perspectives.

## *Vibrio cholerae*, cholera, Robert Koch and the development of Medical Microbiology

In the early period of nineteenth century, cholera was believed to be caused by “miasma” (bad air). In fact, this was one of the reasons which led people to overlook the early work of the Italian scientist Fillipo Pacini who had proposed the germ-theory of cholera and identified the comma shaped organism as the cause of the disease in 1854. In 1883 cholera broke out as an epidemic in Egypt. Fearing that the epidemic might move further and take a grip over Europe, the German government sent a medical team to Egypt which included the German scientist Robert Koch. By the time Koch and his colleagues started their investigation in Alexandria (Egypt), the epidemic started subsiding. This prompted Koch to travel to Calcutta (now Kolkata, India) where the epidemic was still continuing. Investigations carried out with cholera patients led him to identify the comma shaped cholera bacillus (*Vibrio cholerae*, later on named as *Vibrio cholerae Pacini 1854*) as the causative agent of the disease^4^. In an announcement made in 1884, he also claimed the isolation of the organism in pure cultures from the stool of cholera patients, while it was absent in the stool samples from cases with diarrhoea unrelated to cholera. These observations were in conformity with two of the four postulates (known as “Koch’s postulates”) formulated by Koch himself in 1882 to establish the microbial aetiology of infectious diseases^5^. In order to fulfill the criteria laid down in the remaining two of his postulates, Koch tried to infect animals with pure cultures of the organism with little success. He rightly concluded that the animals were not susceptible to cholera and took recourse to the extreme step of infecting himself by drinking pure cultures. However, he came down with only a mild episode of diarrhoea, an outcome which was later on exploited by his opponents to ridicule him. Despite this initial failure, human volunteer studies carried out during the last few decades by various groups of scientists clearly demonstrated that it is possible to induce cholera in humans by oral administration of *V. cholerae* thereby giving further credence to Koch’s postulates, the basic tenets of which hold good even today. One may not be far away from the truth while concluding that the pioneering work of Robert Koch on tuberculosis, anthrax and, subsequently, on cholera laid the foundation stone of Medical Microbiology, a discipline primarily dedicated to the understanding of the cause, mechanism of dissemination as well as control of infectious diseases.

## Cholera, Broad Street pump, John Snow and the science of Epidemiology

In August 1854, cholera broke out in the neighbourhood of London and several hundred people died within a span of couple of days. John Snow, a British physician (anaesthesiologist) who had believed that the disease affecting the gut could be acquired through ingestion of the causative material, immediately grabbed the opportunity to investigate the cases which primarily affected people living in a small area near the Broad Street. After thorough investigation of cases, their location and source of drinking water, Snow came to the conclusion that a pump located in Broad Street had to be the source of cholera^6^. He met the authorities and persuaded them to remove the handle of the pump. Once the handle was removed, cholera cases started declining and the epidemic was soon over. Further investigation revealed that the pump was contaminated with infected material from a nearby sewer which led to the explosive form of the outbreak. The remarkable success of John Snow’s investigation assumes more significance due to the fact that during his life time (1813-1858) neither the causative organism *V. cholerae* was discovered nor the microbial origin of cholera firmly established. As a matter of fact, Snow’s method of investigation of an epidemic based on systematic collection of data on the incidence or number of cases, their temporal as well as geographical distribution patterns, collection of history on individual basis or group-wise and, finally, the use of statistical methodologies for the analyses of data opened up an entirely new approach in medical science that gave birth to the subject of “Epidemiology”.

## Oral redydration solution for the treatment of cholera: A simple solution leading to a major medical discovery in the 20th century

Death in “Asiatic cholera” was attributed to the rapid onset of severe dehydration as a result of massive loss of salt and water in the form of diarrhoea (*cholera gravis*). While administration of saline by the intravenous route, practiced in the nineteenth and twentieth centuries, was able to reduce the mortality rate dramatically, the oral route was found to be ineffective. It was soon evident that patients were not able to absorb sodium chloride when the solution was administered orally. However, there was an imperative need to develop an oral rehydration solution (ORS) based treatment regimen that would help in the clinical management of cases, particularly in the developing world during the time of epidemic. This led scientists and clinicians to vigorously pursue their effort to find a solution to the problem^7^. Finally, a simple yet far reaching observation that glucose added to the ORS was fully absorbed as well as enhanced the absorption of sodium revolutionized the concept of oral rehydration therapy (ORT). It is pertinent to note that the development of the concept and its successful application was an ideal example of partnership between the basic (physiologic) and applied (clinical medicine) research culminating in a discovery that was hailed by the medical journal Lancet as “potentially the most important medical advance in the 20^th^ century”^8^. It is noteworthy that the use of ORT significantly reduced the mortality and morbidity associated not only with cholera but also with certain other dehydrating illness thereby saving the lives of millions of children around the world.

## Cholera toxin (CT): A model protein of considerable biological interest

That cholera is caused by toxic factors released by *V. cholerae* was first proposed by no other person than Robert Koch himself. However, for some reasons, it took several decades before the Indian scientist S.N. De, who workied at Calcutta Medical College, established the role of cholera toxin in the causation of diarrhoea in the rabbit ileal loop model^9^. In the same year (1959), another Indian scientist N. K. Dutta and his colleagues, who were attached to Haffkine Institute at Bombay (now Mumbai), independently reported similar observations^10^. While De used cell-free culture filtrates to induce diarrhoeagenic response in his model, Dutta used cell-free lysates to demonstrate diarrhoea in the infant rabbit model. These discoveries stimulated a lot of interest in the area as a number of scientists were engaged in the purification and characterization of the CT molecule for structure and function studies^11^. Thus, CT was found to be composed of one A subunit and five B subunit polypeptide chains with AB_5_ structure. These studies also revealed that the toxin acts on the target cell by ADP-ribosylation of a G-protein (GTP-binding protein) that locks the membrane bound enzyme adenylate cyclase in an active conformation. Continued activation of the enzyme leads to an elevation in the level of intracellular cAMP thereby resulting into the loss of salt and water in the form of massive diarrhoea. Since its discovery, cholera toxin has served as a useful model to study the mode of action not only of many bacterial toxins but also of certain polypeptide hormones, growth factors and related molecules of considerable biological interest.

## Viable but nonculturable vibrios: An enigma to bacteriologists

Over the years, microbiologists used *in vitro* culture and growth properties of microorganisms as the “gold standard” to demonstrate the presence of viable bacteria in a given specimen. Therefore, the observation made by Xu and coworkers^12^ that *V. cholerae* exists in the environment in “viable but nonculturable” (VBNC) form was initially received with a lot of skepticism by the community. Although there were sporadic reports about the nonculturability of viable bacteria, the VBNC story assumed considerable significance due to its public health importance. In fact, a possible explanation for the failure to isolate *V. cholerae* O1 organisms from the aquatic environment during the inter-epidemic period was attributable to the presence of these bacteria in the VBNC state. While the precise mechanism for the existence of the *V. cholerae* in the VBNC state is yet to be understood fully, possible inducible factors in the environment may include poor availability of nutrient, temperature, salinity, *etc*^13^. Recent studies^14^ have shown that VBNC form of *V. cholerae*, which exists in coccoid shape and retains basal level of metabolic activity, can revert to the culturable and infective form through animal passages. Therefore, the ability of *V. cholerae* to survive in the environment in the viable but nonculturable form in biofilms, and their association with zooplanktons have provided a new dimension to the ecology of *V. cholerae* that has a profound impact on our understanding of the transmission dynamics of cholera.

## *V. cholerae* genome: “A tale of two chromosomes”

Bacterial cells usually contain one circular chromosome which undergoes bidirectional replication for duplication and distribution to daughter cells. Therefore, it was a moment of great surprise when a group of workers demonstrated that the organism *V. cholera*^15^ contained two circular chromosomes in their genome. Whole genome sequence of *V. cholerae* revealed that most of the genes required for the pathogenicity and growth of the organism are located on the large chromosome (chr I) while the small chromosome (chr II) contains genes required for transcriptional regulation and transport of substrates that are important for the assimilation of nutrients from the environment^16^. Distribution of genes in two different chromosomes is believed to facilitate the survival of *V. cholerae* in human intestine as well as in the environment through a complex life cycle as a free living organism and/or in association with biotic or abiotic surfaces. Genomic analyses of *V. cholerae* also led to an intriguing hypothesis of generation of single chromosomal non-replicating cells (“drone cells”) that might aid to the survival of normal (double chromosome) cells under nutrient deficient conditions through secretion of biodegrading enzymes. Thus, *V. cholerae* serves as a model for certain other multichromosomal bacteria that exist in life cycles interacting with diverse microenvironments. Further, experimental data generated so far to understand the mechanism of chromosome replication and segregation in bacteria with divided genomes have been primarily obtained using *V. cholerae* as a model organism^17^.

## Co-ordinately regulated expression of virulence genes in *V. cholerae* and development of the concept of “regulon” in bacterial pathogenesis

In order to survive under different environmental conditions, *V. cholerae* has evolved an intricate signal transduction mechanism that couples environmental stimuli to a complex regulatory network of gene expression or “regulon” through a multi-component regulator system. The work on *V. cholerae* provided a major boost to expand the concept of “operon” (linked genes under the control of a single promoter) to the “regulon” network concept involving multiple genes and operons under the control of a few regulatory proteins. Thus, the co-ordinately regulated expression of a set of virulence genes, which included genes for cholera toxin and colonization pilus, was shown to be triggered by the regulatory cascade under the control of the transmembrane protein ToxR and certain other regulatory proteins, collectively termed as “ToxR regulon”^18^. The availability of the whole genome sequence of *V. cholerae* has allowed researchers to unravel the complexities of the regulon system through microarray based transcriptome analyses of cells isolated either from cholera patients^19^ or from its natural habitat in aquatic environment. Interestingly enough, the concept of co-ordinately regulated virulence gene expression in *V. cholerae* has been found to be a paradigm feature in the pathogenesis of several other bacterial pathogens.

## Bacteriophage, pathogenicity islands and evolution of epidemic causing strains of *V. cholerae*

Although *V. cholerae* strains are of common occurrence in their natural habitat of aquatic ecosystem, not all of these are pathogenic to humans. Out of *V. cholerae* strains belonging to more than 200 serogroups, only the O1 and O139 serogroups are known to be associated with major outbreaks and epidemics of cholera. In recent years, the mechanism of the evolution of pathogenic strains of *V. cholerae* has been an area of intense research. The recently emerged O139 strain^20^ has been shown to be evolved from an O1 El Tor strain primarily through the exchange of O-antigen biosynthesis genes involving horizontal gene transfer mechanism^21^. Evidences are accumulating that suggest that pathogenic strains evolved from the nonpathogenic progenitors through acquisition of virulence-associated genes or gene clusters that are mobile in nature^22^. These include CTX genetic element encoding the genes for CT and other accessory toxins, vibrio pathogenicity islands (VPIs) responsible for the synthesis and expression of the major type of adhesion pili (toxin coregulated pilus or TCP) and certain other virulence factors, *etc*. The CTX element was shown to be of bacteriophage origin (CTXphi) while TCP acted as its receptor^23^. Interestingly, VPI-1 responsible for the expression of TCP was also claimed to be of another bacteriophage origin (VPIphi), thus raising the intriguing possibility of one bacteriophage acting as the receptor of another phage^24^. However, in absence of convincing data for the existence of VPIphi, one would tend to argue that the VPIs were probably acquired by *V. cholerae* through horizontal gene transfer process, the mechanism of which is yet to be clarified. Nevertheless, it would be pertinent to assume that bacteriophages and pathogenicity islands play an important role in the mobilization and/or acquisition of gene segments encoding virulence traits resulting in the evolution of pathogenic bacteria which include *V. cholerae* as well.

## Climatological changes, El Nino and cholera cycle: A model to study the emergence of climate sensitive infectious diseases

There has been a genuine concern that growing climatic changes as a result of global warming are likely to provide a favourable environment for the propagation of disease-causing organisms that may trigger the emergence or re-emergence of infectious diseases around the world. The first major breakthrough in support of this view came through a retrospective study using data on the seasonal incidence of cholera cases in Bangladesh and climatological changes. Using a mathematical model, collaborating scientists from the USA, UK and Spain predicted that the start of an El Nino event in the equatorial Pacific can lead to a surge in the number of cholera cases 11 months later in Bangladesh located several thousand miles away^25^. However, a more complex model had to be developed for the bimodal distribution of cholera cases in a year that could be linked to seasonal variations in local climate, sea-surface temperatures and increased population of planktons supporting the survival and growth of *V. cholerae*. Since the transmission dynamics of cholera bears some similarities with certain types of vector-borne diseases, it would be of interest to determine the El Nino effect on the outbreaks of diseases like malaria, dengue, *etc*^26^. Information generated through the cholera model, thus, provides an opportunity to study other climate sensitive diseases that might lead to the development of appropriate methodologies linking climatic changes to disease outbreak prediction and control^27^.

## “Live” vaccines are dying (?) while “dead” vaccines are still alive (!): The story of vaccination against cholera

A major emphasis in the area of cholera research was toward the development of a vaccine against the disease. Pioneering work in this regard was done by Louis Pasteur as early as in the year 1880. While working on chicken cholera, Pasteur made a serendipitous observation that led to the development of the concept and methodologies of “attenuation” of virulent organisms and their possible use as “live” vaccines to prevent infectious diseases like cholera, anthrax, rabies, *etc*. Surprisingly, despite Pasteur’s approach of vaccinating chickens with live (attenuated) organism, early vaccines against human cholera were based on dead (inactivated) organisms administered by the parenteral route. These vaccines, though provided partial protection, were of limited efficacies to merit further continuation. It was soon realized that more effective stimulation of mucosal immunity in the gut could be achieved through oral rather than parenteral administration of cholera vaccine. Further, availability of recombinant DNA technology provided the opportunity to develop live, attenuated *V. cholerae* strains as candidate oral vaccines capable of replicating in the intestine and in the process inducing local immunity. During the past few decades, different groups of researchers developed several vaccine strains of *V. cholerae*, primarily through deletion of genes encoding one (B) or both A and B subunits of CT. Unfortunately, majority of these vaccines, when administered orally to human volunteers, produced mild to moderate diarrhoea and, therefore, were unsuitable for large scale use^28^. Further, concern regarding the safety of recombinant vaccines was raised following the observation that the genes encoding CT are carried by a phage that can be acquired and integrated in the genome of nontoxigenic *V. cholerae* strains expressing toxin coregulated pilus or TCP. These and other considerations demanded further modifications of the vaccine strains to eliminate residual diarrhoea as well as the possibility of reversal to the virulent phenotype. Therefore, it is quite understandable that only one live recombinant vaccine (CVD 103-HgR derived from a *V. cholerae* O1 strain) is currently available that reasonably meets the criteria of safety, immunogenicity and effectiveness in human volunteer trials^29^. However, the vaccine failed to show convincing level of protection against cholera in a large field trial conducted in Indonesia^30^. All these developments led to the resurgence of the concept of dead (inactivated) vaccines that may be administrated orally without any apparent reactogenicity or risk of reversion to virulence. One such vaccine developed by a Swedish group consists of killed whole cells of *V. cholerae* with purified B subunit (recombinant) of CT^31^ and the vaccine exhibited reasonably satisfactory level of protection in field trials. Interestingly, a cheap and modified version of this inactivated vaccine without the B subunit, administered orally in a population in Vietnam, was also shown to be quite effective for a period more than a year or so^32^.

The search for an effective and safe cholera vaccine has been a long and frustrating one as the disease received considerable attention of scientists over the span of past several decades. However, the search has generated several important cues that are likely to be of profound importance not only for vaccinologists but also for immunologists in general. These include the demonstration of the importance of mucosal immunity (more precisely mucosal antibodies) in cholera and related gut-associated diseases, development of methodologies for optimum stimulation of the immune response in the gut, the understanding of the homing pattern of lymphocytes connecting mucosal associated lymphoid tissues at distant sites and, finally, the relative importance of antibacterial immunity over antitoxin immunity in cholera and ralated enterotoxigenic enteropathies. More importantly, cholera vaccine provides a classic example of a bacterial vaccine that highlights the merit of the continuing debate on the advantages and disadvantages of a live (attenuated) versus dead (inactivated) vaccine.

## Epilogue-Is cholera a disease of “social inequality”?

Cholera is a preventable disease since it is transmitted through ingestion of contaminated food and water. Unfortunately, despite tremendous advancements made in our understanding of the disease and its causative organism, we have not been able to prevent its spread by providing clean water and sanitation. As a result, the disease continues to haunt people living under poor socio-economic conditions. In fact, the number of countries reporting cholera to the World Health Organization increased by 20 per cent (to 56) in 2004, and case fatality rates increased to 2.3 per cent, with rates in some outbreaks reaching as high as 41 per cent in vulnerable groups^33^. The situation continues to remain grim even today. The reason for this dismal scenario may, to a large extent, be attributed to the lack or unequal distribution of health care facilities amongst people living in parts of Asia, Africa, Latin America, *etc*. In many countries, considerations based on race, ethnic origin, caste, religion, colour, *etc*. led to social inequalities that divided the state into “haves” and “have-nots”. The point is exemplified by Briggs and Briggs in their book entitled “Stories in the time of cholera”^34^, where the authors critically analyzed the events leading to the outbreak of cholera in 1992-93 amongst the backward ethnic groups (“Warao”) in the delta region of the Orinoco River in Eastern Venezuela. The authors described in shocking details the sufferings of the poor people of indigenous origin who were not exposed to the concept of personal hygiene or modern treatment facilities (the so called “unsanitary citizens”) as compared to the more affluent “non-indigenous people” to whom modern health care concepts and facilities were available. (“sanitary citizens”). The racial divide probably facilitated the rapid spread of *V. cholerae* on its arrival to these islands that prompted the authors to conclude that “when germs and race mix, however inadvertently, the result is often fatal”. One may add to this combination the role of political scenario of the affected country as an authoritarian regime often likes to cover up the failure of its inefficient (or even corrupt) administrative machinary thereby allowing the continuation of the epidemic unabated. The recent outbreak of cholera in Zimbabawe in 2008 is a glaring example of utmost callousness shown by such a regime in the face of an ongoing epidemic that affected a large part of the country putting half of its population at risk^35^. Fortunately, there is often a silver lining amidst the dark scenario in the form of commendable efforts put forward by the media, social workers and various aid agencies to bring the plight of the people to the attention of international community as well as to initiate relief and other health care measures to alleviate sufferings of the poor people.
